# Accuracy of physician and nurse predictions for 28-day prognosis in ICU: a single center prospective study

**DOI:** 10.1038/s41598-023-49267-y

**Published:** 2023-12-12

**Authors:** Yanxia Huang, Renjing Zhang, Yunxin Deng, Mei Meng

**Affiliations:** Department of Critical Care Medicine, Ruijin Hospital Affiliated to Shanghai Jiao Tong University School of Medicine, Shanghai, 201801 China

**Keywords:** Diseases, Health care, Risk factors

## Abstract

The proportion of correctly predicted prognoses and factors associated with prediction accuracy are unknown. The objective of this study was to explore the accuracy of physician and nurse predictions of 28-day mortality in the ICU. This was a prospective observational single-center study. All medical staff in the ICU have access to patient data, can communicate with patients or clients, and can independently predict the prognosis of patients within 24 h of patient admission. The only question of the questionnaire survey was: What is the patient’s outcome on day 28 (alive or death)? There were 2155 questionnaires completed by 18 physicians and 1916 submitted by 15 nurses. In the 312 patients included, the 28-day mortality rates were predicted by physicians and nurses. The overall proportion of correct prognosis prediction was 90.1% for physicians and 64.4% for nurses (*P* = 0.000). There was no significant difference in the overall correct proportion and average correct proportion among physicians with different seniority levels. The overall correct proportion and average correct proportion increased among nurses with seniority. Physicians in the ICU can moderately predict 28-day mortality in critically ill patients. Nurses with a seniority of less than 10 years in ICU cannot accurately predict 28-day mortality in critically ill patients. However, the accuracy of nurses’ prediction of patients’ 28-day prognosis increased with their seniority in the ICU.

## Introduction

The estimated prognosis of critically ill patients in the ICU often determines their treatment decisions and actual prognosis of patients^1–3^. When a patient is admitted to the ICU, the patient or the patient’s family and physician are equally concerned about whether the patient can leave the ICU alive. The accurate prediction of mortality in critically ill patients is necessary, especially in resource-limited situations.

In addition to clinical scoring systems for predicting mortality, previous studies have shown that clinicians can accurately predict the prognosis of critically ill patients based on initial examinations without scoring systems^4–9^. In fact, prognostic predictions of the outcomes of critically ill patients are frequently conducted and inevitably provide guidance in treatment decisions in daily clinical practice.

However, the accuracy of the estimations was influenced by different factors for the medical staff. Prior studies have analyzed factors related to discordance in prognosis between physicians and surrogate decision makers of critically ill patients and the discriminative accuracy of physician and nurse predictions^10,11^. However, these studies did not provide accurate prognostic accuracy. They did not determine whether predictive accuracy was related to the seniority of the medical staff.

In this prospective study, we explored whether seniority of the nurse or physician was related to predictive prognostic accuracy.

## Methods

This single-center prospective study was conducted from July 22, 2018, to February 22, 2019, in a well-equipped general ICU with 22 beds. Data were collected within 24 h of patient’s admission to the ICU. This observational study was approved by the Ethics Committee of Ruijin Hospital North (2018-004-1). All methods were performed in accordance with the relevant guidelines and regulations.

The participants were physicians and nurses who volunteered to participate in this study. Informed consent was obtained from all subjects. All medical staff included in this study had access to patient data, communicated with patients or agents, and independently predicted the prognosis of patients. The duration of working years of the medical staff was collected. The only question of the questionnaire survey was: What is the patient’s outcome on day 28 (survival or death)?

The patients’ clinical variables were collected, including demographic characteristics (age, sex, body weight, and height), source department, acute physiology and chronic health evaluation (APACHE) II score, sequential organ failure assessment (SOFA) score, Glasgow score, and blood lactate level at the time of admission to the ICU. Whether the patient was transferred to the ICU immediately after surgery. Length of ICU stay. Patient outcomes in the ICU and in 28-day. Each medical staff member’s correct proportion of predicted prognosis was calculated. The average correct proportion was the average of each person’s correct proportion. According to the seniority of medical staff, physicians and nurses were divided into three subgroups: over 10 years, 5–10 years, and less than 5 years in the ICU after graduation from school.

The primary outcome was the correct proportion of prognosis prediction and whether the accuracy of the medical staff’s prediction of patients’ prognosis at 28 days was related to seniority. The second outcome was whether the accuracy of prediction prognosis at 28 days was related to seniority in patients with APACHE II scores > 14^12^.

### Statistical analysis

All statistical analyses were performed using Statistical Package for Social Sciences 23.0 (SPSS, Inc., Chicago, IL, USA). Continuous data were expressed as mean ± standard deviation or median (25–75% interquartile range), and categorical data were expressed as the number of cases (*n*) and percentage (%). The unpaired Student’s *t*-test was used to analyze continuous variables. The chi-squared (*χ*^2^) test was used to compare categorical data. Statistical significance was set at *P* < 0.05.

### Ethical approval

This observational study was approved by the Ethics Committee of Ruijin Hospital North (2018-004-1).

## Results

A total of 312 patients were admitted to the ICU from July 22, 2018, to February 22, 2019. There were 4071 completed questionnaires, 2155 completed questionnaires from physicians and 1916 from nurses. The median age of the patients was 65 years (interquartile range, 50–77), and 184 (59%) were men. Two hundred and six patients were transferred to the ICU after surgery, and 82 and 124 were admitted to the ICU after emergency and non-emergency surgery, respectively. Most patients came from the emergency department, neurosurgery, general surgery, orthopaedic surgery, and cardiac surgery. The average APACHE II score was 12 (interquartile range, 9–18). The mean SOFA score was 5 (interquartile range, 3–8). The duration of ICU stay was 6 (3–13) days. All 312 patients had a 28-day mortality rate of 15.1%. Two hundred sixty-seven patients were alive when they left the ICU (Table 1).

**Table 1:** Patients' characteristics.

Characteristics	Patients, No. (%)
Number	312
Age, median(IQR), years	65 (50–77)
Male	184 (59.0)
Height (cm)	165 (160–170)
Weight (kg)	61(55–70)
BMI (kg/m^2^)	23.0 (20.7–25.5)
Number of patients transferred after surgery	206 (66.0)
Emergency Surgery	82 (26.3)
Nonemergency Surgery	124 (39.7)
The department patients come from	
Emergency department	73 (23.4)
Neurosurgery	72 (23.1)
General surgery	57 (18.3)
Orthopaedic surgery	44 (14.1)
Cardiac surgery	26 (8.3)
Thoracic surgery	17 (5.4)
Urology	4 (1.3)
Neurology	4 (1.3)
Cardiology	3 (1.0)
Hematology department	3 (1.0)
Gastroenterology dept.	3 (1.0)
Geriatrics	3 (1.0)
Respiratory medicine	2 (0.6)
Renal medicine	1 (0.3)
APACHE II score, median(IQR)	12 (9–18)
SOFA score, median(IQR)	5 (3–8)
GCS score, median(IQR)	15 (8–15)
Lactate (mmol/L)	2.2 (1.4–3.4)
Duration of ICU stay, median(IQR),day	6 (3–13)
28-day mortality	15.10%
ICU outcome	
Survival	267 (85.6)
Die	45 (14.4)

There were 20 physicians and 60 nurses in the ICU. The predictions were made by 18 physicians and 15 nurses. The average age of the physicians was 30 (interquartile range, 27–36), and 10 of them were male and 8 were female. Five physicians had worked in the ICU for over 10 years. Three physicians were 5–10 years in the ICU. Ten physicians had less than five years in the ICU since graduation from medical school (Table 2). The average age of the nurses was 28 years (interquartile range, 24–39), and only one of them was male and 14 were female. The number of nurses after over 10 years, 5–10 years, and less than 5 years in the ICU after graduation from nursing school were 3, 4, and 8, respectively.

**Table 2:** Physicians and nurses characteristics.

Characteristics	Physicians no. (%)	Nurses no. (%)
Number	18	15
Age, median (IQR), years	30 (27–36)	28 (24–39)
Sex		
Male	10 (55.6)	1 (6.7)
Female	8 (44.4)	14 (93.3)
Seniority, years		
> 10 years	5 (27.8)	3 (20.0)
5–10 years	3 (16.7)	4 (26.7)
< 5 years	10 (55.6)	8 (53.3)

The overall correct proportion of prognosis predictions was 90.1% for physicians and 64.6% for nurses. The overall correct proportion was significantly higher in physicians than in nurses (*P* = 0.000). The average correct proportion was significantly higher in physicians than in nurses (86.7% and 62.7%, respectively; *P* = 0.000). Physicians and nurses were divided into three subgroups according to seniority of the medical staff. There was no significant difference in the overall correct proportion and average correct proportion among medical staff with seniority over 10 years (90.3% and 92.7%, *P* = 0.227; and 87.6% and 92.7%, *P* = 0.318, respectively). The overall correct proportion and average correct proportion were significantly higher in physicians than in nurses with a seniority of less than 10 years (Table 3).

**Table 3:** Prognosis prediction for all patients.

Characteristics	Physicians	Nurses	*P* value
All patients			
Right	1941	1233	
Wrong	214	683	
Overall correct proportion	90.1%	64.4%	0.000
The average correct proportion	86.7%	62.7%	0.000
Seniority			
> 10 years			
Right	588	391	
Wrong	63	31	
Overall correct proportion	90.3%	92.7%	0.227
The average correct proportion	87.6%	92.7%	0.318
5–10 years			
Right	463	377	
Wrong	51	235	
Overall correct proportion	90.1%	61.6%	0.000
The average correct proportion	89.7%	60.5%	0.000
< 5 years			
Right	890	465	
Wrong	100	417	
Overall correct proportion	89.9%	52.7%	0.000
The average correct proportion	84.8%	52.6%	0.000

**Figure 1:**
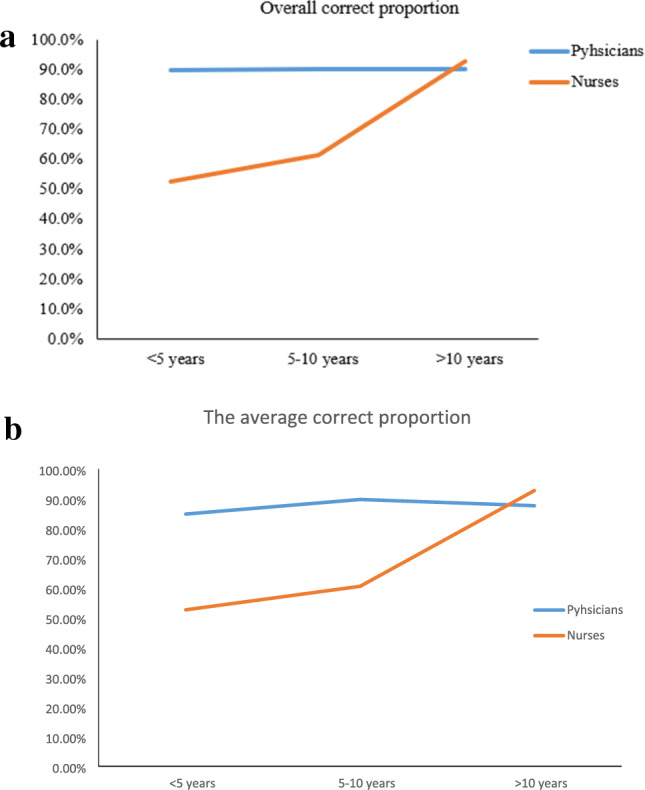
(**a**) The overall correct proportion in physicians and nurses. There was no significant difference between the three subgroups in physicians with overall correct proportion (*P* = 0.961). The overall correct proportion increased with seniority in nurses (*P* = 0.000). (**b**) The average correct proportion in physicians and nurses. There was no significant difference between the three subgroups in physicians with average correct proportion (*P* = 0.824). The average correct proportion increased in nurses with seniority (*P* = 0.000).

To evaluate whether the proportion of correct answers increased with seniority in physicians, three subgroups of physicians were analyzed. The results showed that there was no significant difference between the three subgroups in the overall correct proportion and average correct proportion (*P* values were 0.961 and 0.824, respectively) (Fig. 1a and b). But the subgroup analysis in nurse was quite different. The results showed that the overall correct proportion increased in nurses with seniority (*P* = 0.000), and the average correct proportion increased with seniority (*P* = 0.000) (Fig. 1a and b), respectively.

To evaluate whether the correct proportion was increased in physicians with seniority in patients with APACHE II scores > 14, patients with APACHE II scores of 15 or more than 15 were analyzed (Table 4). The results showed that there was no significant difference between the three subgroups in the overall correct proportion and average correct proportion (*P* values were 0.632 and 0.815, respectively).

**Table 4:** Prognosis prediction for patients’ APACHE II score over 14 in physicians.

	Time since graduation from medical or nursing school, years			*P* value
	> 10	5–10	< 5	
Right	209	166	276	
Wrong	57	43	86	
Overall percentage	78.5%	79.4%	76.2%	0.632
The average percentage	77.9%	79.40%	73.0%	0.815

## Discussion

In this prospective study, we found that the proportion of correct answers was significantly higher in physicians than in nurses. However, the accuracy of physicians’ prediction of patients’ 28-day prognosis did not increase with ICU seniority. The accuracy of nurses’ prediction of patients’ 28-day prognosis increased with their seniority in the ICU. With seniority of over 10 years working in the ICU, physicians and nurses had similar overall correct proportions and average correct proportions.

ICU patients are characterized by severe illness and high mortality rates. Each time a patient is admitted, all medical staff actively rescues the patient. At the same time, everyone has their own opinion on the patient's outcome^13^. The patient's outcome is not only related to the patient's disease state and complications, but also to the expectation of the patient's family members and economic conditions^14^. Previous studies have shown that ICU physicians pay different attention to non-cancer patients, controlled cancer patients, and non-controlled cancer patients^15,16^. This indicates that physicians predict patient prognosis and lead to corresponding differences in medical behavior. Medical staff prediction greatly influences the decision-making of patients or agents^17^. The accuracy of the medical staff prediction prognosis has not yet been reported. The prediction of prognosis in China has not yet been reported.

In this study, all medical staff had the same chance of communicating with patients or their family members to understand the expectations and financial situation of the patient or family member. Patient information was shared equally. The only factor that affected the accuracy of prognosis prediction for patients was the experience of medical staff. In this study, we found that the accuracy of physicians’ prediction of patients’ 28-day prognoses did not increase with seniority. To evaluate the accuracy of physicians’ prediction of patients with severe disease, patients with APACHE II score > 14 were subgroup analyzed. However, these results were negative. Therefore, our results also showed that the accuracy of physicians’ prediction of patients’ 28-day prognosis did not increase with seniority in severely ill patients.

The average correct proportion of predicted prognosis was 86.7% in physicians, and the proportion decreased to 75.9% in patients with an APACHE II score > 14. This suggests that physicians can only moderately predict 28-day mortality in critically ill patients. This result is similar to that of a previous study^13^. Eline et al., showed that the estimations of all students, nurses, and physicians were correct in 71% of 481 cases. The mean APACHE IV score was 70 ± 31. However, their study was different from ours. They did not provide the APACHE IV scores prior to the questionnaire. They did not analyze the correct rate for each group. They did not analyze the relationship between the correct rate and seniority.

There were 60 nurses in the ICU. However, most were young and had worked for less than five years. Most refused to participate in the survey. Thus, only 15 nurses were involved in this study, and the proportion of correct responses was low. This result is similar to that reported by Eline M.’s study^13^. Nurses with less seniority attends to the patient at his bedside every day and is required to perform various treatments^18^. They have little time to assess the patient's condition. Nurses with more seniority, especially those who have worked for more than 10 years, mostly become nursing group leaders. They often take care of patients with less severe illness, while mentoring junior nurses and making comprehensive assessments of patients. They can detect abnormalities in patients early. They are constantly learning to comprehensively analyze the patient's condition. In the process, they continue to improve their judgment.

The proportion of correct answers was significantly higher in physicians than in nurses in this study. These results are similar to those of a previous study^6^. Doctors and nurses have different learning systems. And they have different concerns when treating patients. Physicians evaluated the patient’s condition daily based on symptoms, signs, laboratory tests, and imaging examinations. They can evaluate patients continuously and dynamically. Nurses spend more time on patient beds, but they spend a lot of time checking medical orders, executing medical orders, and keeping track of extensive paperwork, especially for nurses with less seniority. They have little time to analyze a patient's condition and assess the overall state of the patient. The nurse did not actively check the patient's laboratory test results and did not understand how to analyze the image. This may be the reason why prognoses made by physicians are superior to objective models^6–9,18^. This may explain why physicians were more accurate than nurses.

## Limitation

First, this was a single-center questionnaire survey with a small sample size. It can only demonstrate the accuracy of our ICU medical staff in predicting patient prognosis. Second, there were 60 nurses in our department; however, only 15 nurses were involved in this study. Most of them declined to participate in this study because they worried about being unable to predict patient prognosis due to their short working years. This study may have selection bias. A multicenter study could be conducted to confirm the results of this study in future. Third, we only predicted the prognosis of patients at 28 days but did not predict the long-term prognosis of patients. It is unknown whether the accuracy of long-term prognostic prediction increases with seniority. Finally, this is a well-equipped ICU. The results of this study do not represent areas with poor medical resources.

## Conclusion

Physicians in the ICU can moderately predict 28-day mortality in critically ill patients. Nurses with a seniority of less than 10 years in ICU cannot accurately predict 28-day mortality in critically ill patients. However, the accuracy of nurses’ prediction of patients’ 28-day prognosis increased with their seniority in the ICU.

## Data Availability

It will be available from the corresponding author on reasonable request.
